# Asymmetric inheritance of RNA toxicity in *C. elegans* expressing CTG repeats

**DOI:** 10.1016/j.isci.2022.104246

**Published:** 2022-04-11

**Authors:** Maya Braun, Shachar Shoshani, Joana Teixeira, Anna Mellul Shtern, Maya Miller, Zvi Granot, Sylvia E.J. Fischer, Susana M.D. A. Garcia, Yuval Tabach

**Affiliations:** 1 Department of Developmental Biology and Cancer Research, Institute for Medical Research Israel-Canada, Hebrew University of Jerusalem, Jerusalem 9112102, Israel; 2 Institute of Biotechnology, HiLIFE, University of Helsinki, Helsinki 00790 Finland; 3 Division of Infectious Diseases, Boston Children’s Hospital, Harvard Medical School, Boston, MA 02115, USA

**Keywords:** Molecular biology, Molecular genetics, Developmental biology

## Abstract

Nucleotide repeat expansions are a hallmark of over 40 neurodegenerative diseases and cause RNA toxicity and multisystemic symptoms that worsen with age. Through an unclear mechanism, RNA toxicity can trigger severe disease manifestation in infants if the repeats are inherited from their mother. Here we use *Caenorhabditis elegans* bearing expanded CUG repeats to show that this asymmetric intergenerational inheritance of toxicity contributes to disease pathogenesis. In addition, we show that this mechanism is dependent on small RNA pathways with maternal repeat-derived small RNAs causing transcriptomic changes in the offspring, reduced motility, and shortened lifespan. We rescued the toxicity phenotypes in the offspring by perturbing the RNAi machinery in the affected hermaphrodites. This points to a novel mechanism linking maternal bias and the RNAi machinery and suggests that toxic RNA is transmitted to offspring, causing disease phenotypes through intergenerational epigenetic inheritance.

## Highlights

• Maternal origin of expanded CUG repeats induces RNA toxicity in *Caenorhabditis elegans* offspring
• Offspring of affected hermaphrodites show molecular and phenotypic disease phenotypes
• The RNAi machinery is directly related to the maternal inheritance of RNA toxicity
• Altering the RNAi machinery in affected hermaphrodites rescues toxicity in offspring

## Graphical abstract

**Figure:**
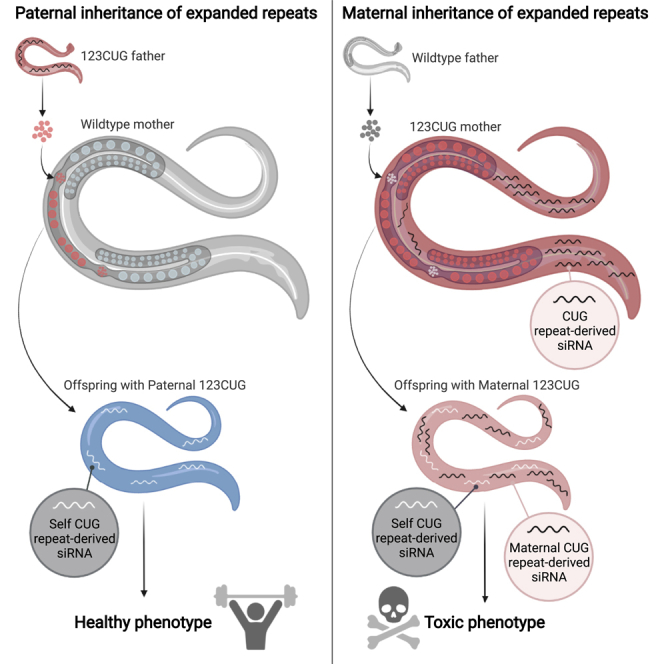

## Introduction

Expansions of short nucleotide repeat sequences in the genome cause over 40 genetic diseases (Paulson, 2018). RNAs transcribed from these expanded regions lead to RNA toxicity and disrupt cellular function through gain-of-function or loss-of-function type of mechanisms (Taneja et al., 1995; Davis et al., 1997; Daughters et al., 2009; Swinnen et al., 2018; Swinnen et al., 2020). Several repeat-based disorders exhibit a correlation between disease severity and gender of the transmitting parent, also known as the parent-of-origin effect (Day et al., 2000; Guilmatre and Sharp, 2012; Echenne and Bassez, 2013).

Several disorders show a maternal bias, including myotonic dystrophy type 1 (DM1), the most common form of adult-onset muscular dystrophy (Ashizawa et al., 1994; Harper, 2009). DM1 is caused by an unstable CTG repeat expansion in the 3′ untranslated region (3′UTR) of the *DMPK* gene (Turner and Hilton-Jones, 2010). Congenital DM1, associated with long expansions and the most severe form of the disease, is characterized by early-onset of symptoms that appear during pregnancy. The newborns suffer from life-threatening complications that include severe generalized weakness and respiratory insufficiency, with up to 40% mortality (Echenne and Bassez, 2013). Contrary to expected deterioration overtime, symptoms improve during childhood but then develop during adolescence until adult-onset DM1 symptoms appear (Ho, 2015).

Congenital myotonic dystrophy is almost exclusively of maternal transmission (Harper and Dyken, 1972; Lanni and Pearson, 2019). Several explanations have been proposed for this maternal bias such as upstream CpG methylation (Steinbach et al., 1998; Yanovsky-Dagan et al., 2015; Barbé et al., 2017; Thomas et al., 2017), but none can account for the different observed disease phenotypes such as the abnormal disease course (Thyagarajan et al., 1991; Cobo et al., 1995; Poulton et al., 1995; Rudnik-Schöneborn et al., 1998; Morales et al., 2015; Yanovsky-Dagan et al., 2015; Lanni and Pearson, 2019). Additional expansion repeat disorders present an early-onset form with distinct clinical features, including Huntington’s Disease-like 2 (HDL2) and spinocerebellar ataxia type 8 (SCA8) (Day et al., 2000; Margolis et al., 2005; Schneider et al., 2012). Both are caused by CUG repeats in the 3′UTR, suggesting a common mechanism driven by the toxic CUG RNAs.

RNA interference (RNAi) is a conserved gene-silencing mechanism (Fire et al., 1998). The enzyme Dicer processes double-stranded RNA (dsRNA) into short-interfering RNA (siRNA) of ∼21 nucleotides. These form a protein-RNA complex that degrades and inhibits translation of the target messenger RNA (mRNA) bearing a complementary sequence (Hammond, 2005). Expanded DNA repeats, when transcribed, form stable hairpins that accumulate as RNA foci in the nucleus and cytoplasm (Taneja et al., 1995; Mizielinska et al., 2013; Garcia et al., 2014; Kumar et al., 2017). Evidence from nematodes (Qawasmi et al., 2019), drosophila (Yu et al., 2011), and mammals (Krol et al., 2007; Cappella et al., 2018; Murmann et al., 2018) show that Dicer can target these dsRNAs and cleave them to siRNAs (Krol et al., 2007; Bañez-Coronel et al., 2012; Rué et al., 2016; Cappella et al., 2018) that cause global changes in gene expression (Qawasmi et al., 2019). The RNAi machinery can mediate heritable epigenetic modulations, a phenomenon termed RNAi inheritance (Alcazar et al., 2008; Rechavi et al., 2011; Ashe et al., 2012; Shirayama et al., 2012; Buckley et al., 2012; Gu et al., 2012; Luteijn et al., 2012; Marré et al., 2016; Houri-Zeevi and Rechavi, 2017; Rechavi and Lev, 2017; Perales et al., 2018; Ewe et al., 2020). siRNAs are inherited from parent to progeny and cause transgenerational gene silencing (Marré et al., 2016; Lev et al., 2019; Ewe et al., 2020; Lev and Rechavi, 2020). In mammals, the presence of small RNAs from maternal origin was shown in human and mouse cord blood (Miura et al., 2010; Ouyang et al., 2014; Li et al., 2015; Luo et al., 2018; Su et al., 2020).

Here, we use *Caenorhabditis elegans* to uncover mechanisms that underlie the parent-of-origin RNA toxicity effect. The RNAi machinery is highly conserved across eukaryotes (Tabach et al., 2013), making *C. elegans* ideal to study epigenetic inheritance (Rechavi et al., 2011; Houri-Ze’evi and Rechavi, 2016; Houri-Zeevi and Rechavi, 2017) and the RNAi machinery (Timmons and Fire, 1998; Ketting et al., 1999, 2001; Tabara et al., 1999; Grishok et al., 2000; Grishok et al., 2001; Knight and Bass, 2001; Sijen et al., 2001; Kim et al., 2005; Fischer et al., 2008) in a controlled isogenic background. As patient cell lines are inadequate for investigating parental effects, nematodes provide an excellent whole-organism alternative. We recapitulate a maternal bias in a *C. elegans* model expressing expanded CUG repeats and uncover the requirement of the RNAi pathway in the affected mothers to enhance toxicity in progeny. Conversely, disrupting the RNAi pathway in mothers bearing expanded RNAs rescued toxicity in offspring.

## Results and discussion

### Maternal inheritance of CUG repeats enhances toxicity phenotypes

**Figure 1:**
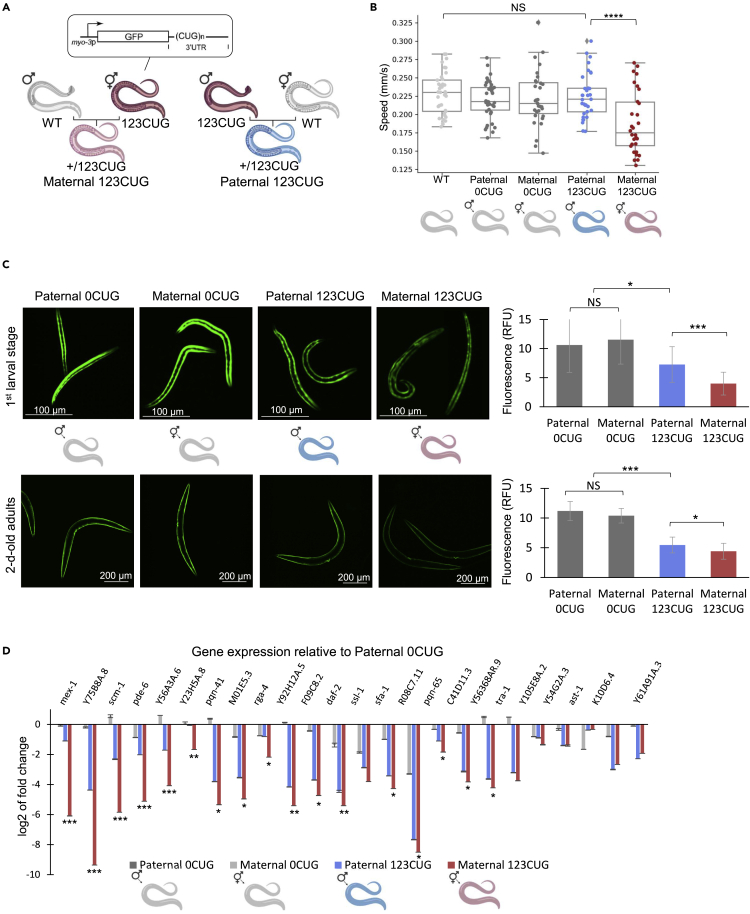
Maternal inheritance of repeats aggravates disease phenotype (A) *C. elegans* model for repeat expansions and a general scheme of the parent-of-origin experimental system. 123 CUG repeats are expressed in the 3′UTR of GFP under the myo-3 promoter. (B) Motility assay for assessment of parent-of-origin effects. Moving speed of WT, paternally inherited 0CUG, maternally inherited 0CUG, paternally inherited 123CUG, and maternally inherited 123CUG nematodes (n = 45). The average of three biological replicates is represented. Data are represented as a mean ± SD of three biological replicates and significance was calculated using an ANOVA test followed by post-hoc two-tailed Student’s t-tests. (C) Fluorescence levels of Maternal 0CUG, Paternal 0CUG, Maternal 123CUG, and Paternal 123CUG nematodes in the first larval stage and two-day-old adults. Relative fluorescence was computationally quantified (n = 50) and representative fluorescent microscopy images are shown. Data are represented as a mean ± SD of three biological replicates and significance was calculated using an ANOVA test followed by post-hoc two-tailed Student’s t-tests. (D) Gene expression fold change of 24 genes bearing ≥7 CTG/CAG repeats in two-day-old adults, relative to Paternal 0CUG. The qPCR is an average of three biological experiments and three technical replicates. ∗p < 0.05, ∗∗p < 0.01, ∗∗∗p < 0.001, ∗∗∗∗p < 0.0001; NS - not significant. See also Figures S1–S4 and S7.

To investigate the mechanisms underlying the parent-of-origin toxicity effect associated with CUG repeat expansions, we used *C. elegans* strains expressing GFP containing either 0 or 123CTG repeats (0CUG and 123CUG, respectively) in the 3′UTR, under the regulation of the *myo-3* muscle-specific promoter (Figure 1A) (Garcia et al., 2014; Qawasmi et al., 2019). As we have previously shown, the toxicity of the expanded repeats causes impaired motility and higher susceptibility to heat stress (Figures S1) (Qawasmi et al., 2019). To examine whether toxicity caused by expanded repeats is differentially expressed if inherited from maternal versus paternal lines, we crossed 123CUG males with wild type hermaphrodites (Paternal 123CUG) and 123CUG hermaphrodites with wild-type males (Maternal 123CUG) (Figure 1A). We then assessed the severity of the toxicity in the progeny and compared the nematodes with paternal inheritance of CUG repeats with nematodes with maternal inheritance.

Motility assays of two-day-old nematodes showed that the average moving speed of nematodes with maternally inherited repeats is significantly lower (p < 0.0001, Figure 1B) than the Paternal 123CUG or the control groups: WT, Paternal 0CUG, and Maternal 0CUG. To examine that it is not a strain-specific effect we repeated the experiment in independent 0CUG and 123CUG strains (GR3208 and GR3207, respectively), which showed a similar maternal enhancement of toxicity (Figure S3). However, in this strain, Paternal 123CUG shows a significant speed reduction as well. Moreover, the Maternal 123CUG nematodes were more susceptible to heat stress as compared with Paternal 123CUG (Figure S4). Additional asymmetry was observed in nematode morphology. Maternal 123CUG nematodes were significantly shorter at the first larval stage, with an average length of 173.2 versus 187.5 μm in the Paternal 123CUG and 0CUG animals (p = 1.83∗10^−6^). A significant but attenuated difference in length was also observed in two-day-old adults (p = 0.002, Figure S4).

**Figure 2:**
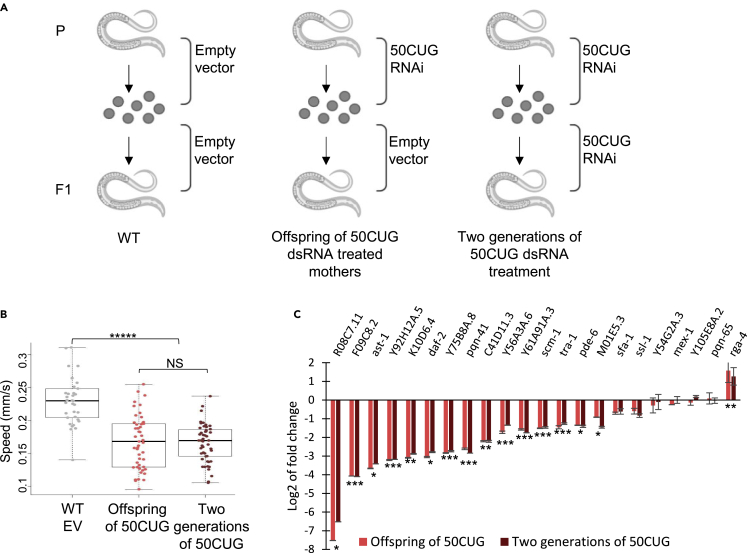
Feeding nematodes with 50CUG dsRNA recapitulates toxicity phenotypes (A) Experimental scheme: Wild-type nematodes were fed dsRNA bearing 50CUG repeats for one or two generations. (B) Motility assay of F1 two-day-old adult WT, offspring of WT nematodes treated with 50CUG dsRNA, and WT nematodes fed 50CUG dsRNA for two generations (n = 45). Data are represented as a mean ± SD of three biological replicates and significance was calculated using an ANOVA test followed by post-hoc two-tailed Student’s t-tests. ∗∗∗∗∗p < 0.00001, NS - not significant. (C) Gene expression fold change of 24 genes bearing ≥7 CTG/CAG repeats in two-day-old adults as determined by qPCR for 50CUG treatment groups relative to wild type. Three biological and three technical replicates were analyzed for this experiment. ∗p < 0.05, ∗∗p < 0.01, ∗∗∗p < 0.001.

The maternal effect was also observed at GFP fluorescence levels. 123CUG animals had a noticeable decrease in GFP protein levels with age as compared to 0CUG (Garcia et al., 2014; Qawasmi et al., 2019). Activation of the RNAi machinery by the 123CUG repeats seems to be responsible for the decreased fluorescence (Qawasmi et al., 2019). We measured and compared the fluorescence levels of Maternal 123CUG, Paternal 123CUG, Maternal 0CUG, and Paternal 0CUG nematodes. The Maternal 123CUG animals exhibited a stronger decay in fluorescence as compared to the Paternal 123CUG animals (Figure 2C), while the Maternal 0CUG and Paternal 0CUG strains did not differ. Interestingly, this difference was more pronounced for nematodes at their first larval stage and slightly diminished overtime (Figures 2C and S4). Notably, 123CUG nematodes did not show changes in repeat size in successive generations, thus the Maternal and Paternal 123CUG nematodes used in our experiments share the same genetic background (heterozygous 123CUG). In both groups, the repeats were targeted by the RNAi machinery and processed to siRNAs (Figure S2).

To conclude, these data showed that the phenotypes associated with toxicity were asymmetrically inherited and enhanced in the Maternal 123CUG animals.

### Maternal inheritance of repeats suppresses gene expression of genes bearing endogenous CTG- and CAG-repeats

Across species, transcribed CUG- and CAG-repeats trigger abnormal silencing of genes bearing complementary short repeat sequences, a hallmark of silencing by the RNAi machinery (Krol et al., 2007; Cappella et al., 2018; Qawasmi et al., 2019). To determine whether the RNAi pathway also played a role in the preferential inheritance of RNA repeat toxicity by the offspring of Maternal versus Paternal 123CUG nematodes, we looked for changes in gene expression that could be linked to the CUG repeats and the RNAi machinery. We measured the expression levels of 24 genes containing seven or more endogenous CTG/CAG repeats that we previously validated as markers of CTG-targeted silencing in two-day-old adult nematodes (Qawasmi et al., 2019). The expression of these genes was enriched in muscles, neurons, hypodermis, and intestine, correlative to the disease phenotypes (Qawasmi et al., 2019). The elevated expression of small repeat-derived RNA and coordinated suppression of genes bearing complementary sequences was evident in both 123CUG groups but significantly enhanced in the Maternal 123CUG animals (Figures 1D and S2). These data showed that the transcriptomic changes associated with expanded CUG repeats also present a maternal bias.

### Directly activating the RNAi pathway by feeding nematodes 50CUG dsRNA recapitulates toxicity phenotypes

To better characterize the mechanism and establish that the maternal bias results from the repeat-derived small CUG RNAs, and depends on the siRNA pathway, we adopted a complementary approach. We used RNAi, a very common silencing approach in *C.elegans*, to directly trigger the RNAi machinery by exogenous dsRNA bearing expanded CUG repeats. We fed wild type animals with bacteria expressing RNAs bearing 50CUG repeats (50CUG RNAi) and assessed three groups (Figure 2A): nematodes grown on an empty vector (EV), offspring of hermaphrodites (F1) fed 50CUG dsRNA, and nematodes fed 50CUG dsRNA for two generations. The nematodes from both intervention groups, two generations of 50CUG dsRNA-treated animals (P0 and F1 were treated), and offspring of treated animals (F1 were not treated), all exhibited impaired motility with an average moving speed of 0.17 mm/s versus 0.23 mm/s in the wild-type group (p = 1.18∗10^−10^, Figure 2B). Both 50CUG dsRNA-treated groups recapitulated the suppression in the expression of the 24 CTG/CAG-containing genes (Figure 2C). Similar impairment was observed in the offspring of 50CUG dsRNA-treated mothers and the nematodes that were directly treated with dsRNA containing expanded repeats. Hence, the feeding of repeat-bearing dsRNA recapitulates the intergenerational RNA toxicity caused by endogenous expression of expanded repeats. This suggests that the motility impairment and the changes in gene expression are at least partly mediated by the RNAi machinery.

### Altering the RNAi machinery in 123CUG hermaphrodites rescues toxicity in offspring with maternally inherited repeats

**Figure 3:**
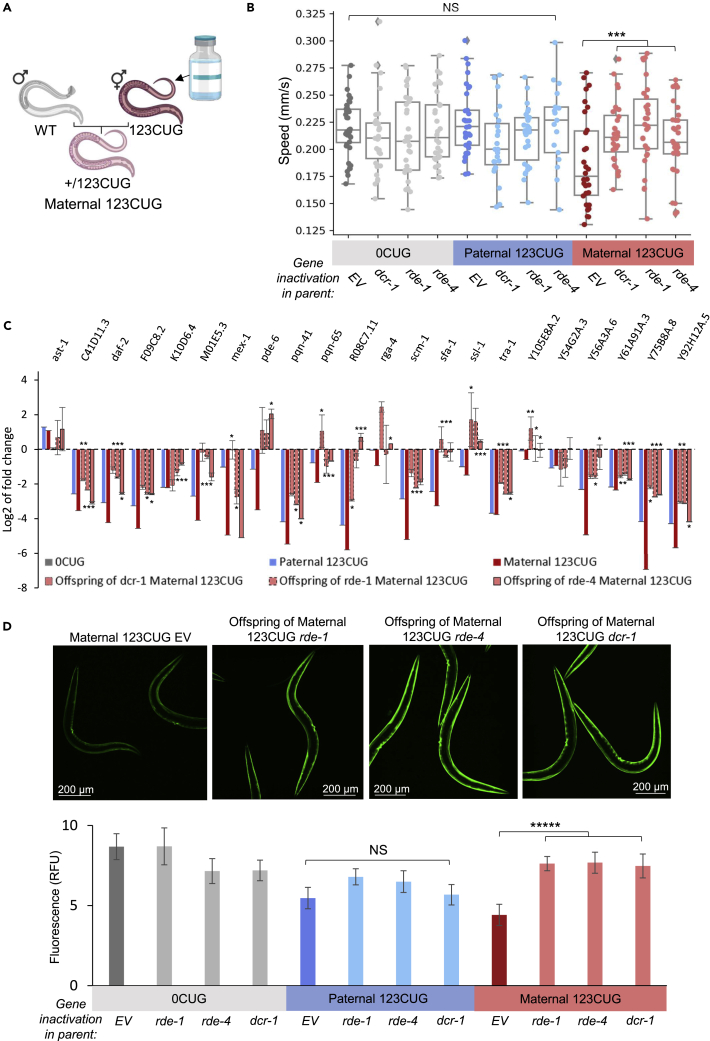
Exclusive treatment of affected 123CUG mothers rescues phenotype in offspring with maternally inherited repeats (A) Scheme of the intervention: treatment of 123CUG hermaphrodites using RNAi against *dcr-1, rde-4,* and *rde-1,* until the fourth larval stage, followed by mating with N2 males to create Maternal 123CUG animals. Offspring were observed for phenotypic effects. (B) Motility assay of the two-day-old adult progeny of mothers treated against *rde-4, dcr-1,* and *rde-1* (n = 45). Data are represented as a mean ± SD of three biological replicates and significance was calculated using an ANOVA test followed by post-hoc two-tailed Student’s t-tests. (C) Gene expression fold change of 24 genes bearing ≥7 CTG/CAG repeats, determined by qPCR for offspring following RNAi treatment to mothers, relative to 0CUG. Three biological and three technical replicates were analyzed for this experiment. (D) Fluorescence levels of two-day-old adult Maternal 123CUG offspring subsequent RNAi treatment of mothers. Relative fluorescence was computationally quantified (n = 50). Representative fluorescent microscopy images of two-day-old adults are shown. Data are represented as a mean ± SD of three biological replicates and significance was calculated using an ANOVA test followed by post hoc two-tailed Student’s t-tests. ∗p < 0.05, ∗∗p < 0.01, ∗∗∗p < 0.001, ∗∗∗∗∗p < 0.00001, NS - not significant. See also Figures S5 and S6.

Maternally enhanced intergenerational toxicity is dependent on repeats being generated in the mothers either by feeding (50CUG dsRNA) or expression of a transgene (Maternal 123CUG) (Figures 1 and 2). We assumed that partial disruption of the RNAi pathway in affected mothers has the potential to revert the phenotypes in the F1 progeny. As complete loss-of-function of key players in the RNAi machinery can cause severe phenotypes (Denli et al., 2004; Fischer et al., 2013), we used RNAi to regulate components in the RNAi machinery. This is a well-validated approach that partly silences proteins in the pathway, thus reducing the steady state of the RNAi machinery but with limited effects on the phenotype (Kim et al., 2005; Tabach et al., 2013). A complete knockout of major factors in the RNAi machinery like *dcr-1* or *rde-4* resulted in impaired motility phenotypes (Figure S5) whereas knockdown using RNAi (Kim et al., 2005; Tabach et al., 2013) did not produce an obvious detrimental effect (Figure 3).

We fed 123CUG hermaphrodites *rde-1, rde-4,* and *dcr-1* double-stranded RNAs to reduce processing of toxic RNAs into siRNAs. At the fourth larval stage (L4) we stopped the RNAi treatment. We removed the hermaphrodites from the RNAi plates, crossed them with wild-type males on EV plates and assessed the offspring for toxicity effects (Figure 3). Timing the RNAi feeding restricted the silencing of the RNAi machinery components to the 123CUG parent hermaphrodites (Marré et al., 2016). We observed that the progeny of 123CUG mothers with suppressed RNAi machinery displayed significantly improved movement with an average speed of 0.22 mm/s versus 0.19 mm/s in Maternal 123CUG (Figure 3B). A significant improvement in response to heat stress was also observed (Figure S6). Expression levels of CTG/CAG-bearing genes were downregulated to a lesser extent in the offspring of the RNAi-treated mothers as compared to the empty vector control (Figure 3C)*.* Fluorescence levels were on average 171% higher in the offspring of Maternal 123CUG nematodes that were treated with RNAi of *rde-1, rde-4,* and *dcr-1* in comparison to Maternal 123CUG animals (Figure 3D). Importantly, this effect is tightly associated with the maternal inheritance bias as Paternal 123CUG nematodes were not different from the WT worms (Figures 3B and 3D).

**Figure 4:**
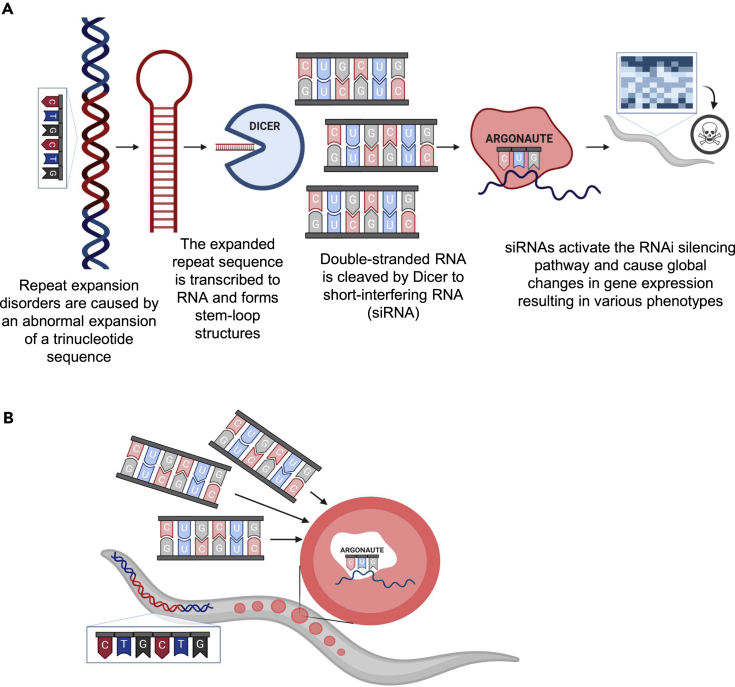
Proposed mechanism (A) The RNAi machinery mediates RNA toxicity in expansion repeat disorders. (B) Repeat-derived siRNAs from affected mothers initiate early RNAi-induced toxicity in offspring.

To conclude, downregulating siRNA production in 123CUG hermaphrodites rescued pathogenic phenotypes in their offspring (Maternal 123CUG), but the analogous treatment of 123CUG males did not affect their offspring (Paternal 123CUG). These data imply that the parent-of-origin effect is mediated by maternal repeat-derived siRNAs that enhance early gene silencing in their progeny (Figure 4). These results point to a potential therapeutic approach for repeat-carrying mothers to ameliorate disease phenotype in progeny.

Epigenetic inheritance of small RNA is established (Houri-Zeevi and Rechavi, 2017; Rechavi and Lev, 2017; Lev and Rechavi, 2020), but a role for this inheritance in RNA toxicity or a link to expanded repeats and maternal bias was unknown as well as its potential therapeutic impact in this context. Here, we demonstrated how the repeated RNAs can cause maternal bias through the RNAi machinery and that most of the disease phenotypes in offspring may be rescued by targeting the RNAi machinery in the mothers. Overall, we characterized in *C. elegans* complex molecular crosstalk between DNA repeats, RNA toxicity, the RNAi machinery in gender-specific toxicity inheritance, and its consequences for disease onset and progression.

Regarding the limitations of the 123CUG system, it could be argued that the intergenerational effect that we described is partly because of transgene silencing induced by the repetitive arrays, thus not specifically triggered by the presence of the expanded repeats. However, we believe it unlikely to have a major effect on the 123CUG strains. Transgene silencing of somatic tissues is rare (Fire et al., 1991; Knight and Bass, 2002) and the 0CUG and 123CUG strains were specifically generated as complex arrays, where the plasmids containing the CUG repeats were diluted 1 to 100^th^ in digested genomic DNA in the generation of the transgenes. These complex arrays are known to be less prone to germline transgene-silencing (Kelly et al., 1997; Nance and Frøkjær-Jensen, 2019). To address this concern, we crossed the 123CUG nematodes with *adr-1; adr-2* mutants and found no change in motility (Figure S7). *adr-1* and *adr-2* encode the *C. elegans* ADARs, adenosine deaminases acting on dsRNA. These genes have been shown to act on repetitive transgenes: by editing dsRNA derived from these transgenes, silencing by the RNAi pathway is prevented. The ADARs and RNAi compete for the same substrate, transgene dsRNA. If the 123CUG was subject to transgene silencing, loss of ADARs would have led to increased transgene-silencing through an increase in dsRNA entering the RNAi pathway, resulting in more severe toxicity phenotypes (Knight and Bass, 2002). Accordingly, these results support our proposed mechanism that the toxic generation of siRNAs is derived from the hairpin structures of the expanded repeats and is specific to these structures.

Despite the evolutionary distance between *C. elegans* and mammals, many cellular and molecular mechanisms are conserved (Lai et al., 2000). Specifically, the RNAi machinery is highly conserved across eukaryotes (Tabach et al., 2013) and in *C. elegans* (Rechavi et al., 2011; Houri-Ze’evi and Rechavi, 2016; Houri-Zeevi and Rechavi, 2017). Thus, utilizing *C. elegans* provides a controlled isogenic background and offers a fast way to screen the effect of RNA toxicity at the molecular and phenotypic level. The fact that we were able to recapitulate a maternal bias and other RNA toxicity phenotypes in *C. elegans,* suggests that our proposed mechanism is potentially relevant to patients. Specifically, it may offer a new way to investigate the high risk (64%) for congenital disease in offspring born to mothers carrying over 164 CTG repeats (Morales et al., 2015).

The human genome consists of over 800 genes bearing endogenous 6CTG repeats. In humans, endogenous RNAi activity was reported in both germline and somatic cells (Diallo et al., 2003; Yi et al., 2003; Svoboda, 2014), and small RNAs originating from the mother were shown to infiltrate the embryo and affect global gene expression, thus regulating their progeny’s cellular processes during development (Ng et al., 2013; Li et al., 2015; Vilella et al., 2015; Gross et al., 2017). We speculate that some of the toxic repeat-derived siRNAs from the DM1-affected mother can reach their progeny during pregnancy; hence, mis-regulating normal development. At birth, the additional flux of repeat-derived siRNAs from the mother is interrupted, thus likely contributing to the improvement observed in the symptoms in the first years. Importantly, healthy progeny (expanded repeat-free) are also exposed to the maternal repeat-derived siRNAs, which offers a potential explanation for the relatively high incidence of the described minor anomalies (Martorell et al., 2007). Although maternal biases were characterized in DM1 and SCA8 (Day et al., 2000), distinct CNG repeat expansions have been shown to form secondary structures and be Dicer targets (Krol et al., 2007), suggesting a conserved role for RNA interference in RNA toxicity transgenerational gene silencing.

Over 20 expansion repeat disorders are characterized by RNA toxicity (Todd and Petrucelli, 2018). Most of these diseases are extremely rare and are frequently hard to diagnose. Even if maternal effects play a role in these diseases this could be easily missed. For example, the maternal bias in DM1 was first described over 60 years after Steinert characterized the disease (Harper and Dyken, 1972). Hence, targeted research may identify additional CNG disorders presenting parent-of-origin effects.

Finally, understanding the parent-of-origin effect in repeat disorders and potentially additional diseases presenting similar effects may have an enormous impact on the way we currently treat these disorders. Therefore, future research should investigate the distinct contributions through RNA interference and transgenerational gene silencing of different repeat expansions to pathogenesis. Those approaches would provide an opportunity to develop novel disease-modifying therapeutics for DM1 and possibly other expansion repeat disorders.

### Limitations of the study

Here, we offer a new mechanism for the intergenerational toxicity of CUG repeats in a *C. elegans* system. Although it is tempting to assume that the phenotypic similarity and the conservation of the RNAi machinery across eukaryotes suggests that homologous mechanisms can be found in other species, further studies are needed to confirm the mechanism in mammals. Further work should aim to examine the repeat-derived siRNAs in human patients and specifically in pregnant women that give birth to babies with congenital myotonic dystrophy.

## STAR★Methods

### Key resources table

**Table:** 

REAGENT or RESOURCE	SOURCE	IDENTIFIER
**Bacterial and virus strains**		

*Escherichia coli* OP50	CGC	WB Cat# WBStrain00041969
*Escherichia coli* HT115 transformed with the L4440 vector	Ahringer's RNAi library	WB Cat# WBStrain00041079

**Experimental models: Organisms/strains**		

*C. elegans*: Strain: N2	CGC	WB Cat# WBStrain00000001
*C. elegans*: Strain: GR2024	Gary Ruvkun's lab	WB Cat# WBStrain00043193
*C. elegans*: Strain: GR2025	Gary Ruvkun's lab	WB Cat# WBStrain00043194
*C. elegans*: Strain: GR3208	Gary Ruvkun's lab	N/A
*C. elegans*: Strain: GR3207	Gary Ruvkun's lab	N/A
*C. elegans*: Strain: BB22	CGC	WB Cat# WBStrain00000440
*C. elegans*: Strain: PD8753	CGC	WB Cat# WBStrain00030623
*C. elegans*: Strain: BB21	CGC	WB Cat# WBStrain00000439

**Oligonucleotides**		

Primers for RT-qPCR, see Table S1	This paper	N/A

**Software and algorithms**		

GraphPad Prism 9.0	GraphPad Prism Software, Inc	https://www.graphpad.com/
Micam 2.4	Developed by Marien van Westen	https://micam.software.informer.com/
MakeAVi	Developed by John Ridley	http://makeavi.sourceforge.net/
DeltaPix	DeltaPix Software, Inc	https://www.deltapix.dk/
ImageJ	Schneider et al. (2012)	https://imagej.nih.gov/ij/

### Resource availability

#### Lead contact

Further information and requests for resources and reagents should be directed to and will be fulfilled by the lead contact, Yuval Tabach (yuvaltab@ekmd.huji.ac.il).

#### Materials availability

This study did not generate new unique reagents.

### Experimental model and subject details

#### *C. elegans* and RNAi strains

*C. elegans* strains GR2024 (*mgls64[myo-3p::gfp::3′utr123(CUG)] III*), GR3207 (*mgIs84[myo-3p::gfp::3′utr123(CUG)])* (termed 123CUG) and GR2025 (*mgls64[myo-3p::gfp::3′utr0(CUG)] V*), GR3208 (*mgIs85[myo-3p::gfp::3′utr0(CUG)])* (termed 0CUG) were used (Garcia et al., 2014). These animals express 123CUG or 0CUG repeats in the 3′UTR of GFP in the body wall muscle cells under the *myo-3* promoter. The N2 (Bristol) strain was obtained from the *Caenorhabditis* Genetics Center (Minneapolis, USA) and used as a wild-type strain. For the RNAi mutant assays, strains BB22 (*adr-2(gv42) rde-4(ne299) III) (rde-4* mutant*),* and PD8753 (*dcr-1(ok247) III/hT2 [bli-4(e937) let-?(q782) qIs48] (I;III)*) *(dcr-1* mutant*)* were obtained from the Caenorhabditis Genetics Center. For the ADAR experiments, the strain BB21 (*adr-1(tm668) I; adr-2(ok735) III*) was obtained from the *Caenorhabditis* Genetics Center. *C. elegans* strains were handled using standard methods and grown at 20°C unless otherwise indicated (Stiernagle, 2006).

### Method details

#### Crossing Maternal and Paternal 123CUG strains

Ten 123CUG one-day-old males were put on a plate with four L4 wildtype hermaphrodites. Plates were synced after 48 h, eggs were left to hatch overnight in M9, and L1 nematodes with Paternal 123CUG were produced. For Maternal 123CUG, the process was replicated with 10 wildtype one-day-old males and four L4 123CUG hermaphrodites. To rule out the possibility of biased phenotypes due to self-mating of the hermaphrodites, the crosses were validated by two approaches: ∼50% prevalence of males in the F1 offspring was assessed, and randomly selected F1 animals were sequenced for the presence of repeats.

#### Gene inactivation

RNAi-mediated gene inactivation was achieved by feeding nematodes bacterial strains expressing dsRNA as previously described (Kamath et al., 2001). RNAi clones were obtained from the Ahringer’s library (Kamath and Ahringer, 2003). A single colony of RNAi bacteria was grown overnight at 37°C in LB with 100 mg/ul ampicillin, and then seeded onto NGM plates with carbenicillin. Vector expression was induced by adding isopropyl β-D-thiogalactopyranoside (IPTG) to a final concentration of 1 mM directly over the bacterial lawn and left to dry for 24 h. The empty L4440 vector (EV) was used as a negative control.

#### Motility

On day two of adulthood (five days after hatching), five *C.* *elegans* males were picked and placed on 60 mm NGM plates without food. The nematodes were left to recover for 20 min after which they were filmed. Over 15 animals were counted per experiment and the data from three biological replicates were combined. Images were captured using a digital microscope and Micam 2.4 Software. The resolution was 2048 × 1536 pixels and a total number of 120 frames were taken at a rate of one capture per second for 120 s. In each experiment, all images were captured with the same focus, on the same day, and at room temperature. The video was built by MakeAVi software with a playback rate of 15 frames per second. The animals were analyzed using Tracker 5.0 software by defining the tail of the animal as a point mass and manually tracking its position for each frame.

#### Stress assays

Synchronized nematode eggs were placed on NGM plates seeded with RNAi bacteria or OP50 (as indicated) and supplemented with 100 mM IPTG (four mM final concentration). For the heat shock assay, at day one of adulthood 80 animals were transferred onto pre-warmed plates without bacteria (10 animals per plate) and exposed to 35°C. Survival rates were recorded every 2 h.

#### Morphology and length

Maternal and Paternal 123CUG animals were generated and left to hatch in M9 until the first larval stage. Then they were placed on NGM plates seeded with OP50 and immediately imaged using a Nikon SMZ800N Microscope. After 72 h, the nematodes were imaged once again. The nematodes’ length was determined using the DeltaPix software.

#### Target genes for siRNA silencing

A BLAST (Altschul et al., 1990) search was conducted to identify genes with seven or more CTG/CAG repeats, and with no more than two mismatches, that could serve as the most obvious targets for siRNA silencing. Thirty-one transcripts were identified and specific primers for 24 of those genes were generated. Expression levels were determined using RT-qPCR as described below. We were unable to generate specific primers and establish expression levels for the seven remaining genes.

#### RT-qPCR analysis of mRNA and siRNA expression

Total RNA was extracted from the whole body of *C. elegans* nematodes using Trizol Reagent (Ambion, USA) and a NucleoSpin RNA isolation kit (Macherey-Nagel, Germany). Hundreds of nematodes were collected for each experiment. For mRNA, reverse transcription was performed using a cDNA reverse transcription kit (Applied Biosystems, USA), and mRNA expression levels were measured with qPCR. SYBR-Green (Bio-Rad, USA) was used in a CFX-384 Real-Time PCR system (Bio-Rad). For siRNA, reverse transcription was performed using the MystiCq miRNA cDNA synthesis Mix (Sigma Aldrich, USA). MystiCq SYBR green (Sigma-Aldrich) and universal PCR primer (Sigma-Aldrich) were used for RT-qPCR. Data were analyzed using the ΔΔCt method. Relative quantities of gene transcripts were normalized to *rpl-32* and *cdc-42* for the mRNAs and *mir-46-3p* for the siRNAs. All primers used in this research were designed using the NCBI Primer Blast (sequences depicted in Table S1).

#### Fluorescence

Maternal 123CUG, Paternal 123CUG, and 0CUG nematodes were grown on empty vector bacteria and images were taken at the first larval stage and two-day-old adults. The RNAi groups were treated as previously described and imaged at the same ages. The animals were washed twice with M9, anesthetized using 10 mM sodium azide (Sigma Aldrich, USA), and placed on an agar pad. Images were taken using a Spinning-Disk confocal microscope. For all fluorescence images, the images shown within the same figure panel were collected using the same exposure time and then processed identically in ImageJ.

The Figures and graphical abstract were created with BioRender.com.

### Quantification and statistical analysis

The statistical analysis for the motility assays was performed using an ANOVA test followed by post-hoc pairwise testing with a two-tailed Student’s *t* test, α = 0.05. Statistical analyses for the heat stress assays were performed using log-rank (Mantel-cox) and Gehan-Breslow-Wilcoxon tests, α = 0.05. Statistical analyses for the morphology and length assays were performed using a two-tailed Student’s *t* test, α = 0.05. Statistical analyses for the fluorescence assays were performed using the ANOVA test followed by post-hoc pairwise testing with a two-tailed Student’s *t* test. The statistical details of experiments can be found in the figure legends.

## Data Availability

• All data reported in this paper will be shared by the Lead contact upon request. • This paper does not report original code. • Any additional information required to reanalyze the data reported in this paper is available from the Lead contact upon request.
