# Supplementary material for: Asymmetric inheritance of RNA toxicity in C. elegans expressing CTG repeats
Source: iScience. 2022 Apr 11;25(5):104246. doi: 10.1016/j.isci.2022.104246 (PMC9051633; doi:10.1016/j.isci.2022.104246)
Supplement: Document S1. Figures S1–S7 and Table S1 [file mmc1.pdf]

iScience, Volume *25*

Supplemental information

Asymmetric inheritance of RNA toxicity

in *C. elegans* expressing CTG repeats

Maya Braun, Shachar Shoshani, Joana Teixeira, Anna Mellul Shtern, Maya Miller, Zvi Granot, Sylvia E.J. Fischer, Susana M.D. A. Garcia, and Yuval Tabach

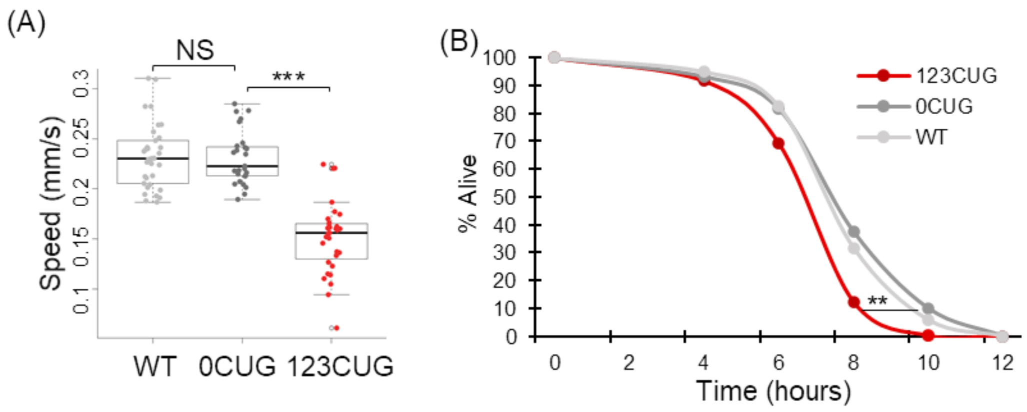

Figure S1: Phenotype expression of homozygous 123CUG nematodes, Related to Figure 1. (A) Motility assay (moving speed) of WT, 0CUG, and 123CUG nematodes (n=60) for two-day-old adults. Data of three biological replicates is represented and significance was calculated using an ANOVA test followed by post-hoc two-tailed Student's t tests. (B) Survival curve of WT, 0CUG, and 123CUG day-one adult nematodes following heat shock (35°C, n=80). The results shown are from a representative experiment of three biological replicates. Statistical analyses were performed using log-rank (Mantel-cox) and Gehan-Breslow-Wilcoxon tests. ** p<0.01; *** p<0.001; NS - not significant.

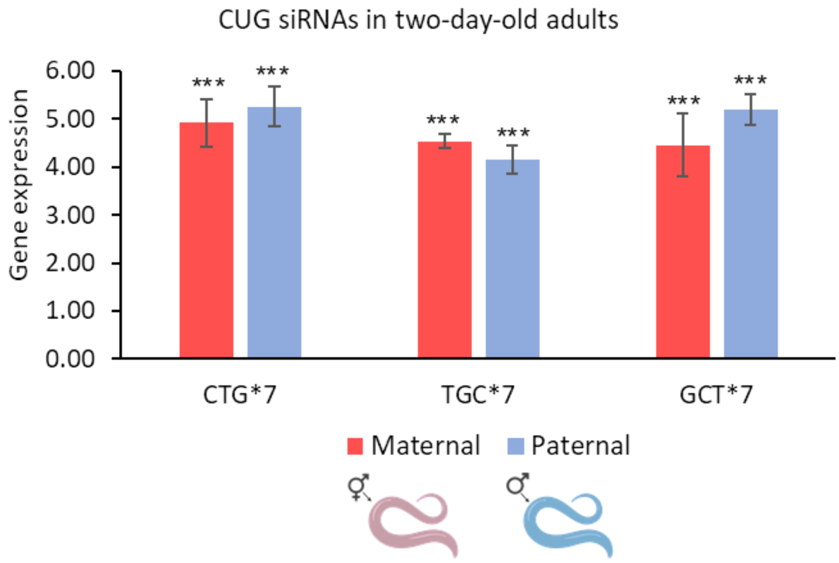

Figure S2: Quantification of CUG-repeat siRNAs by RT-qPCR, related to Figure 1. Enrichment of CUG-repeat siRNAs in two-day-old adult Maternal 123CUG and Paternal 123CUG, relative to 0CUG nematodes. Data corresponds to three primers: CTG*7, TGC*7, and GCT*7. *** p<0.001

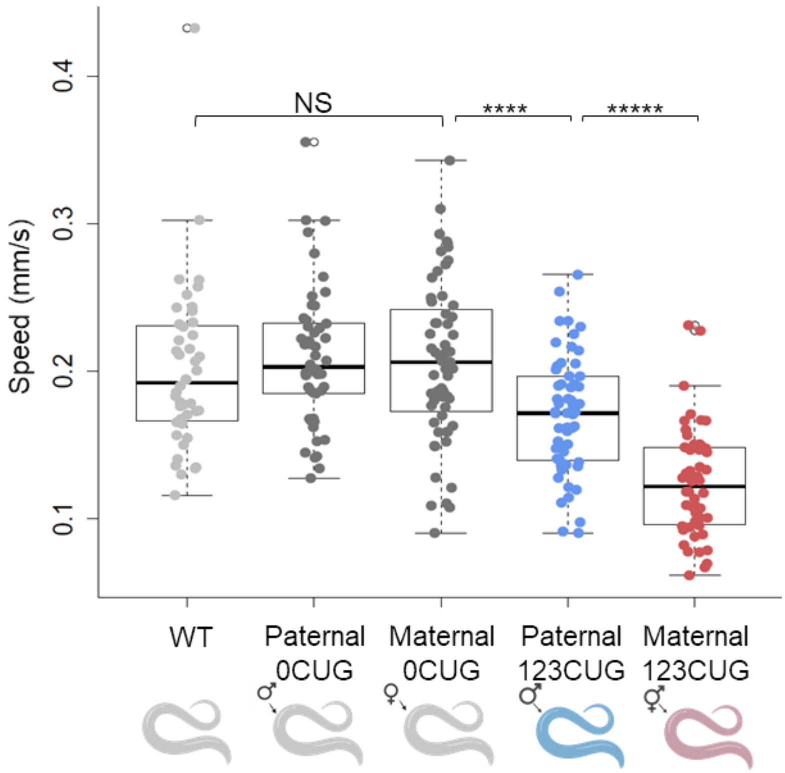

Figure S3: Motility phenotype recapitulated on different 0CUG and 123CUG strains (GR3207 and GR3208), related to Figure 1**.** Moving speed of WT, paternally inherited 0CUG, maternally inherited 0CUG, paternally inherited 123CUG, and maternally inherited 123CUG nematodes (n=45). Data of three biological replicates are represented. Significance was calculated using an ANOVA test followed by post-hoc two-tailed Student's t tests. **** $p<0.0001$, ***** $p<0.00001$, NS - not significant.

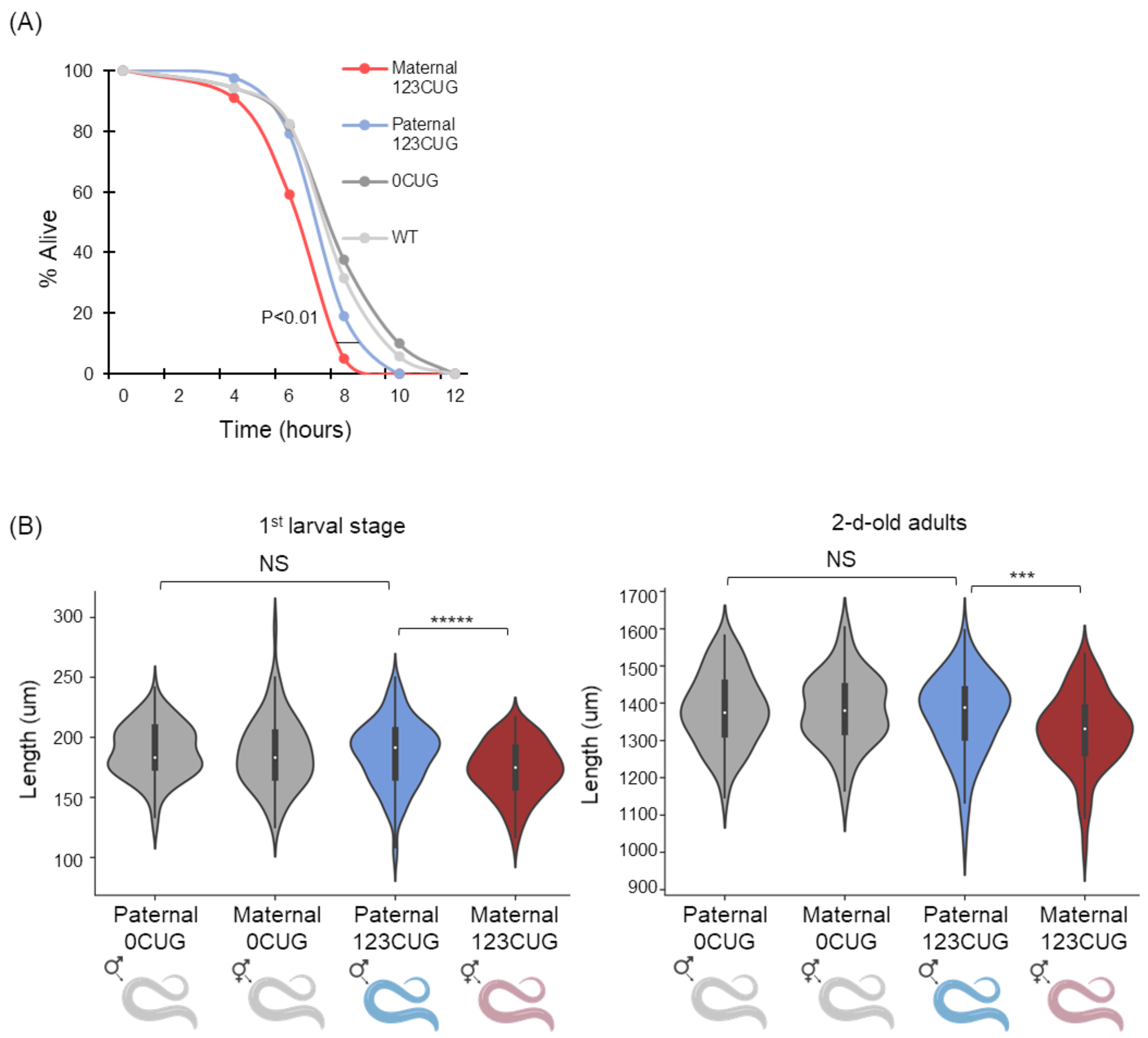

Figure S4: Maternal inheritance of repeats aggravates disease phenotype, related to Figure 1**.** (A) Survival curve of Maternal 123CUG and Paternal 123CUG animals following heat shock (35°C, n=80). The results shown are from a representative experiment of three biological replicates. Statistical analyses were performed using log-rank (Mantel-cox) and Gehan-Breslow-Wilcoxon tests. (B) Maternal origin of expanded repeats reduces nematode size. Violin plot represents length measurements of 120 nematodes for each group. Significance was calculated using an ANOVA test followed by post-hoc two-tailed Student's t tests. *** p<0.001, ***** p<0.00001, NS - not significant

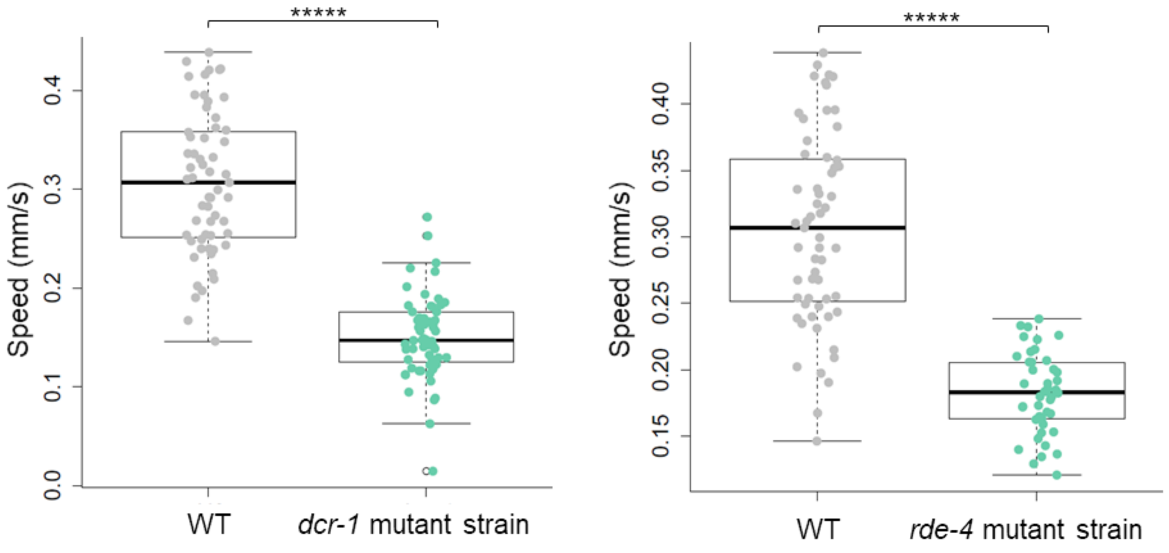

Figure S5: *dcr-1* and *rde-4* knockout mutants present with severely impaired motility, related to Figure 3. Moving speed of mutant strains (n=60). Data of three biological replicates are represented and significance was calculated using a two-tailed Student's t test. ***** p<0.00001.

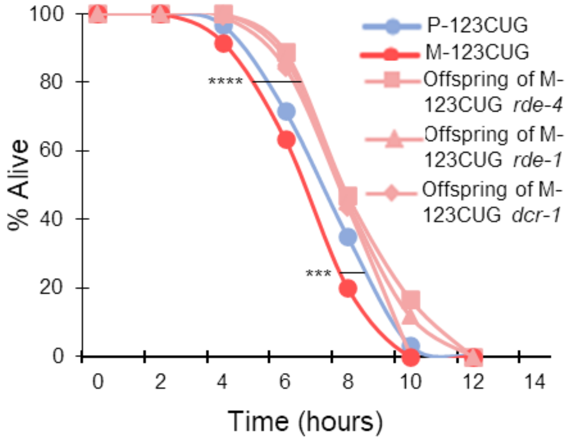

Figure S6: Related to Figure 3. Survival curve under heat shock (35°C) of one-day-old adult progeny of treated mothers. The results shown are from a representative experiment of three biological replicates. Statistical analysis was performed using log-rank (Mantel-cox) and Gehan-Breslow-Wilcoxon tests (n=80).

M-123CUG, Maternal 123CUG; P-123CUG, Paternal 123CUG. ** p<0.01, **** p<0.0001

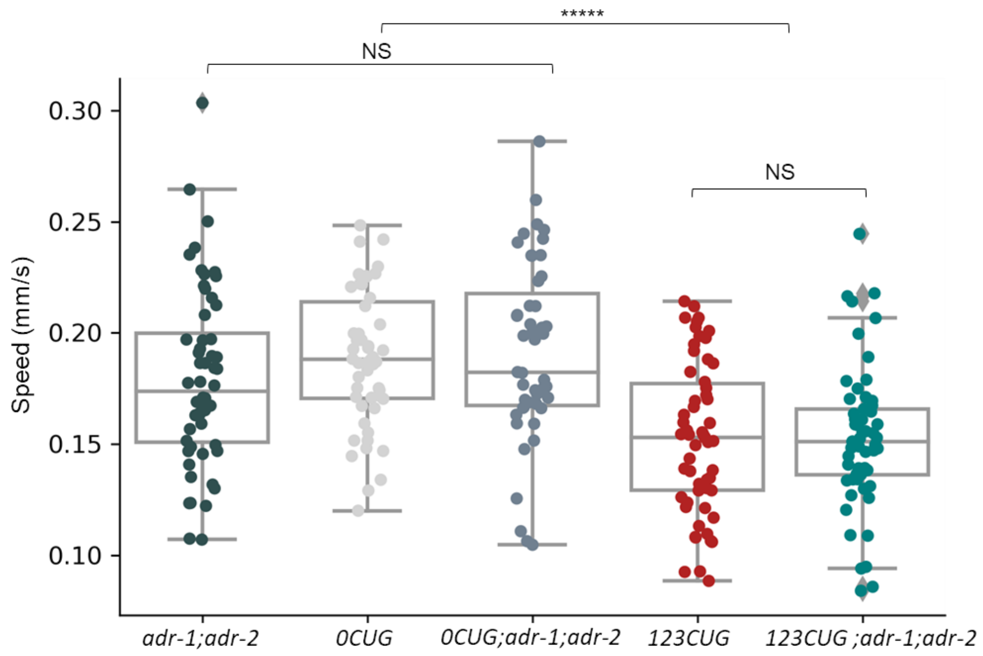

Figure S7: Loss of ADARs does not affect toxicity phenotype in 123CUG nematodes, related to Figure 1**.** Motility assay (moving speed) of *adr-1;adr-2,* 0CUG*, 0CUG;adr-1;adr-2,* 123CUG and *123CUG;adr-1;adr-2* nematodes (n=60) for two-day-old hermaphrodite adults. Data of three biological replicates is represented and significance was calculated using an ANOVA test followed by post-hoc two-tailed Student's t test. ***** p<0.00001, NS - not significant

Table S1: Primer sequences for qPCR, related to STAR Methods.

(A) Primers for qPCR of genes bearing CTG repeats

Gene		
ast-1	Forward Primer	5'-AGTGGCTCGAAAATGGGGAG-3'
	Reverse Primer	5'-AGCCTGTGCGATTCCTTGAA-3'
rga-4	Forward Primer	5'-ACACTCTTGGAGAGGTATGCT-3'
	Reverse Primer	5'-TTCCTTGCGGAAAATCCCGT-3'
**Y75B8A.8**	Forward Primer	5'-AACATTCCCACAGGACTCGG-3'
	Reverse Primer	5'-GAAGATTGCTCTGGGGACGA-3'
ssl-1	Forward Primer	5'-AGCCAGGATGCGAAGATTGA-3'
	Reverse Primer	5'-GTTCGATTTTGAGCCGTTCG-3'
**R08C7.11**	Forward Primer	5'-ATGGTCGGAGGGTGTCTTCT-3'
	Reverse Primer	5'-GTCTGCGTCGTGTACTCCTT-3'
**Y23H5A.8**	Forward Primer	5'-TCGTGTGGAACGACCATCTG-3'
	Reverse Primer	5'-ATCCTGGTCCCTTGGTCCAT-3'
mex-1	Forward Primer	5'-AAAACCAGAGAACATTCGCACC-3'
	Reverse Primer	5'-CGACGAGCAGTGTATCCTCC-3'
pqn-41	Forward Primer	5'-CGCATACCAGTGACACCGAA-3'
	Reverse Primer	5'-CGGAATTCCTCATCCCTCCG-3'
pqn-65	Forward Primer	5'-CGACGAATCGGCTGAAGTACA-3'
	Reverse Primer	5'-GTCCTGCGACTGCTCCTAAG-3'
**Y105E8A.2**	Forward Primer	5'-GACTTATCAGGTTTGCCACGG-3'
	Reverse Primer	5'-CATTTTCGATTCGGCGGGTC-3'

daf-2	Forward Primer	5'-AATGCCGAGAGACACGATGC-3'
	Reverse Primer	5'-GGCTTCTTTCCACCGAGAGT-3'
**Y53G8AR.9**	Forward Primer	5'-TTTATCATCCGTCCGAGGCG-3'
	Reverse Primer	5'-CTGGCACCCTCGATTCTGAT-3'
**Y92H12A.5**	Forward Primer	5'-TCTGTTTCGAACGGGCTCTC-3'
	Reverse Primer	5'-GGCGCTTCTCATTACCCCAA-3'
**F09C8.2**	Forward Primer	5'-AATCTGAAACCGGAGCAGCA-3'
	Reverse Primer	5'-AGTTTGGTGTGAGCAGAGGAG-3'
**Y61A9LA.3**	Forward Primer	5'-GCTCTGGAGACTGGCTACAA-3'
	Reverse Primer	5'-CTTTGATCTCCTGGTCCCGC-3'
**C41D11.3**	Forward Primer	5'-TGGACAAGGCAGTTCGTTGG-3'
	Reverse Primer	5'-GTGTTGAGCACGACTTCTCG-3'
**K10D6.4**	Forward Primer	5'-TTCACCGAATGCACACCGTA-3'
	Reverse Primer	5'-CTTCCGTGGGACACACAAGA-3'
scm-1	Forward Primer	5'-ACCAACCACTCATCAGTCAAC-3'
	Reverse Primer	5'-ACCTGCTGCACTTCTCTGTC-3'
**M01E5.3**	Forward Primer	5'-TATGTATGCTGCTGGGCTGG-3'
	Reverse Primer	5'-ATTCACGGACGTGCACAATG-3'
tra-1	Forward Primer	5'-GCCCAACAAGTGTGAGTATCC-3'
	Reverse Primer	5'-TTCTCCGGTGGGTTTTCAGG-3'
sfa-1	Forward Primer	5'-ATTGCTGAAGCCACTGCTCT-3'
	Reverse Primer	5'-GAGCTCCGTTTGCCAAATCC-3'
pde-6	Forward Primer	5'-ATCGCAAATCGCCAAAGACG-3'
	Reverse Primer	5'-ACGCGTATTCCACGTCACAT-3'
**Y54G2A.3**	Forward Primer	5'-AAAAACCAGCGGATGAGGTGA-3'
	Reverse Primer	5'-TTTGAGAGCTTCTAGTTTGTACGGA-3'
**Y56A3A.6**	Forward Primer	5'-TGTTATGCCACCGCGAAAAG-3'
	Reverse Primer	5'-GAGGTGGCTGATGGTGGAAA-3'
cdc-42	Forward Primer	5'-CTGCTGGACAGGAAGATTACG-3'
	Reverse Primer	5'-CTCGGACATTCTCGAATGAAG-3'
rpl-32	Forward Primer	5'-AGGGAATTGATAACCGTGTCCGCA-3'
	Reverse Primer	5'-TGTAGGACTGCATGAGGAGCATGT-3'

(B) Primers for repeat-derived sRNAs

**MystiCq Universal PCR primer**	Reverse Primer	
**7CTG**	Forward Primer	5'-CTGCTGCTGCTGCTGCTGCTG-3'
**7TGC**	Forward Primer	5'-TGCTGCTGCTGCTGCTGCTGC-3'
**7GCT**	Forward Primer	5'-GCTGCTGCTGCTGCTGCTGCT-3'
mir-46-3p	Forward Primer	5'-TGTCATGGAGTCGCTCTCTTCA-3'
